# Effects of rhodioloside on the neurological functions of rats with total cerebral ischemia/reperfusion and cone neuron injury in the hippocampal CA1 region

**DOI:** 10.7717/peerj.10056

**Published:** 2020-11-09

**Authors:** Yue Zhang, Xinqing Guo, Guohua Wang, Jidan Liu, Peiyu Liang, Huan Wang, Chunyan Zhu, Qiong Wu

**Affiliations:** 1 College of Animal Science and Technology, Northwest A&F University, Yangling, China; 2 Department of Basic Medicine, Heze Medical College, Heze, China; 3 Medical College, Qinghai University, Xining, China; 4 Pharmaceutical Section of Municipal hospital of Heze, Shandong, China

**Keywords:** Rhodioloside, Total cerebral ischemia/reperfusion, Bcl-2/Bax, p53

## Abstract

Rhodioloside, the main effective constituent of *Rhodiola rosea*, demonstrates antiaging and antioxidative stress functions and inhibits calcium overloading in cells. These functions imply that rhodioloside may exert protective effects on hippocampal neurons after total cerebral ischemia/reperfusion injury. In this study, male Wistar rat models of total cerebral ischemia were constructed and randomly divided into four groups: sham-operation, ischemia/reperfusion, low-dosage, and high-dosage groups. The result showed that rhodioloside treatment reduced the apoptosis rates of hippocampal neurons and the histological grades of cone cells in the hippocampal CA1 region, but neuronal density was significantly increased. Besides, the protein expressions of Bcl-2/Bax and p53 were measured and found Bcl-2/Bax was increased and p53 protein level was reduced. Therefore, rhodioloside might have protective effects on rats with ischemia/reperfusion brain injury.

## Introduction

**Figure 1:**
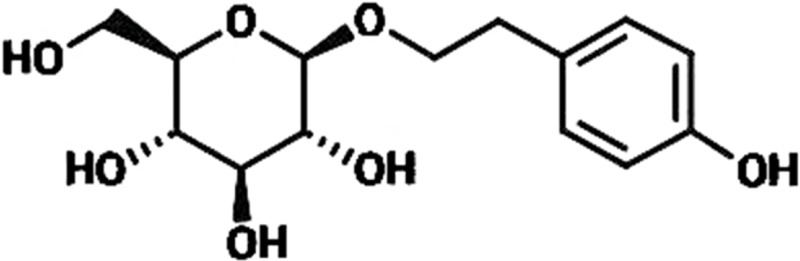
The molecular structure of rhodioloside.

*Rhodiola rosea* (*R. rosea*) is a plant mainly grows in Qinghai-Tibet Plateau and has been extensively applied to produce medicine for anti-hypoxia (Liu et al., 2018; Yan, Xu & Fu, 2018). *R. rosea* is also regarded in traditional Chinese medicine as a good nutrient to regulate the flow of *qi*, nourish blood, promote blood circulation to resolve blood stasis, and enhance brain health; thus, we speculated whether *R. rosea* would play a positive role in treating Ischemic brain injury, which is a clinically common injury with severe effects on human life quality (Kong et al., 2012; Yu, Liang & Yu-feng, 2007; Zhang et al., 2004). In this research, we primarily studied the protective function of the ingredients of *R. rosea* for ischemic brain injury. Rhodioloside is the main effective constituent of *R. rosea*. Its molecular structure is shown in Fig. 1. It has been proved that rhodioloside shows a protective effect on mouse ischemic stroke (Zhang et al., 2018). In this study, rat model of total cerebral ischemia/reperfusion was constructed by the four-vessel occlusion method (Pulsinelli & Buchan, 1988). According to Gao’s thesis (Gao et al., 2012), rhodioloside of 12 mg/kg or 48 mg/kg were selected to observe the effects of rhodioloside on total cerebral ischemia/reperfusion rats. The neurological deficit and hippocampal neuronal injury of rats after total cerebral ischemia/reperfusion were observed and the mechanism underlying the protective effects of rhodioloside was further studied through the expression of Bcl-2/Bax and p53 proteins to provide new evidence and basis for studies on the clinical prevention and treatment of cerebrovascular diseases.

## Material and Methods

### Animal ethics

Healthy male Wistar rats weighing 250–300 g with the same genetic background were provided by the Experimental Animal Center of Heze Medical College. Rats were raised in SPF environment and were free to food and water. Rat bedding was changed every day. At the end of animal experiment, the rats were executed by decapitation, and there was no surviving rat at the end of this experiment. This is a kind of euthanasia for rats suitable in this experiment. All surgical procedures conformed to institutional and national guidelines and were approved by the Animal Care and Use Committee of the Northwest A&F University (GC2018).

### Drugs, reagents, and instruments

Rhodioloside, whose molecular structure is shown in Fig. 1, was provided in lyophilized powder form by the Traditional Chinese and Tibetan Medicine Lab of the Medical College of Qinghai University. Analytically pure thionine was purchased from Sigma, US. SABC-AP immunohistochemical reagent kit, Bcl-2 antibody, Bax antibody, p53 antibody, and total protein quantitative assay kit were all procured from Nanjing Jiancheng Bioengineering Institute. FACS420 FCM was a product of Becton Dickinson Corporation, US.

### Establishment of the total cerebral ischemia rat model

Experimental animals were placed in a rat cage with a padded floor and were allowed to acclimate for a week with sufficient water and feed before establishing the total cerebral ischemia rat model by the four-vessel occlusion method. The concrete method was performed as follows: a healthy male Wistar rat was anesthetized with 10% chloral hydrate (350 mg/kg) through intraperitoneal injection. The exposed atlas transverse process at the neck-side muscle was separated and preheated electrocautery needle with a tip diameter of 0.5 mm was inserted from wing-shaped holes identified under a stereoscopic microscope to coagulate and block bilateral vertebral arteries. Rats with 2-day recovery were selected to construct the total cerebral ischemia model. After the rats were anesthetized via ethyl ether inhalation and regained consciousness, both common carotid arteries were clipped for total cerebral ischemia. The dilation of pupils and absence of the righting reflex during the occlusion of the cephalic arteries indicated that total cerebral ischemia had occurred and it lasted 8 min. The sham operation was performed by following the same process as described above without clipping common carotid arteries. During the operation and total cerebral ischemia process, 1% procaine was applied to the local part of the wound to prevent pain, and room temperature was maintained above 20 °C. An incandescent light bulb was used to maintain their anal temperature at 37 °C until they recovered. After total cerebral ischemia, wounds were sutured, and antibiotic was applied to prevent infection.

### Experimental grouping and drug administration

**Figure 2:**
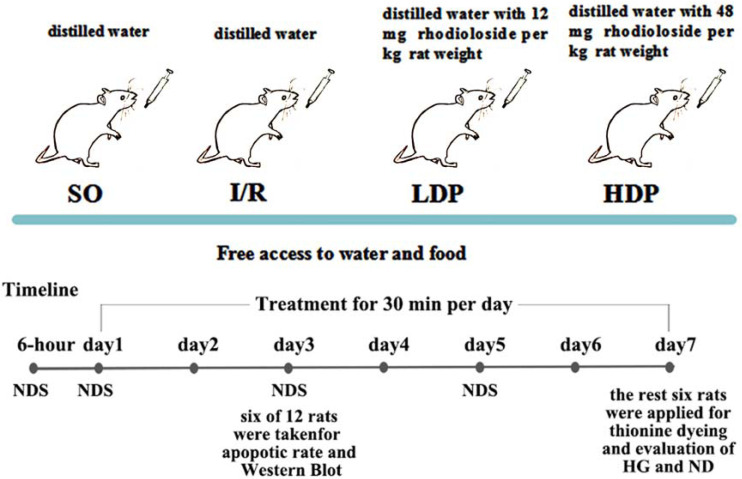
Schematic diagram of experimental design.

The bilateral vertebral arteries of all rats were coagulated and blocked and were randomly allocated into four groups (12 rats for each group): a. the sham operation group (SO), b. the ischemia/reperfusion (I/R) group, c. the I/R + 12 mg/kg rhodioloside group (low-dosage group, LDP), and d. the I/R + 48 mg/kg rhodioloside group (high-dosage group, HDP). Rats in LDP and HDP groups received 12 and 48 mg of rhodioloside per kg rat weight respectively through gavage, while I/R and SO groups received equal quantities of distilled water by the same method. Rhodioloside was dissolved in distilled water according to the weight of rats (1 ml/100 g), and each rat received gavage once a day for 30 min in seven continuous days. The treatment schematic diagram is shown in Fig. 2. Then, six rats were taken at the third day after sham operation or cerebral ischemia surgery to observe the apoptosis rates of hippocampal neurons and to determine the expression level of Bcl-2/Bax and p53 proteins. Kirino, Tamura & Sano (1984) found that neuronal apoptosis induced by I/R often occurs after 24 hr and peaks at 72 hr. Therefore, we selected the third day (72 hr) as a time point to measure neuronal apoptosis. While Nitatori et al. (1995) found that cell death mostly occurs at three to four days after I/R, with the seventh day being the most pronounced, which is called delayed neuronal death. Thus, other six were collected at the seventh day after sham operation or cerebral ischemia surgery to observe Hippocampal CA1 histological grading (HG) and neuronal density (ND) under light microscopy.

### Neurological deficit scoring (NDS)

NDS was performed to evaluate the neurological deficit of rats at different time after reperfusion in reference to the method described by Brambrink et al. (2006). Evaluation items included consciousness, smell, eyesight, respiration, reflex, movement function, balancing ability, exploratory activity, environmental response, and epilepsy attacks. Scores ranged from 0 to 100, wherein a score of 0 indicated the absence of a neurological deficit and a score of 100 indicated the most severe neurological deficit. Scoring was performed by nonexperimental professional persons blinded to the experimental grouping and was strictly conformed to standard for evaluation. With settled standard and professional scorers, reliable results were obtained.

### Cerebral histopathological evaluation

Animal brains were collected after decapitation. Brain tissues were incised at one mm to four mm behind the optic chiasma through the coronary cutting method. The collected samples were fixed in 4% paraformaldehyde and then rinsed with 0.1 mol/L PBS for three times. Tissue slices were then successively dehydrated in 70%, 80%, 90%, 95%, and 100% ethanol for 2 h, 12 h, 1 h, 1 h, and 45 min, respectively. The slices were decolorized in xylene for 3 min and then subjected to 4.5 h of paraffin infiltration at 60 °C for paraffin embedding. Then the collected samples were sectioned by 5 µm in thickness, and dried on APES slides for 24 h in 60 °C. Thionine staining was used to observe histological changes in the hippocampus. HG and ND were determined under an optical microscope to evaluate histological changes in the hippocampal CA1 region in accordance with the method proposed by Kato et al. (1991) and Kitagawa et al. (1991); the evaluation standards are as follows: (1) HG- grade 0, no neuronal death; grade I, some scattered dead neurons are present; grade II, a chunk of dead neurons are present; and grade III, nearly all neurons are dead. Average grades of two sides were taken as statistical values. (2) ND: the number of cone cells with complete cytomembranes, full nuclei, and clear nucleolus in one mm segment of the hippocampal CA1 region were counted, and the values of the three segments in each bilateral hippocampus were taken as statistical values.

### Detection of hippocampal neuronal apoptosis rate

The heads of animals were broken to collect brains after execution. The bilateral hippocampus was separated out on an ice plate after red blood cells were washed off with precooled normal saline. Hippocampal tissues were cut into pieces and rubbed gently using ophthalmological tweezers on a 120-mesh stainless steel net with normal saline washing. Then filter the suspension using a 300-mesh nylon net and centrifugate for 2 min at 500–800 r/min. A 0.1 ml (containing 1 × 10^5^ cells) of the single-cell suspension was taken and mixed with 1 ml of ethidium bromide dye liquor to stain Annexin FITC/PI at 4 °C under dark conditions. Apoptosis detection was conducted using Flow Cytometry (BD FACSAria III, USA) and the results are shown in quadrantal diagrams. We analyzed the percentage in Q2 quadrant, which represents late apoptotic rate.

### Western blot analysis

The ischemia-side hippocampus of the rats were collected in 0.8 ml of cell lysis solution, and blended by an ultrasonic cell disruption instrument under low temperature. Then they were centrifuged at 4 °C and 12, 000 r/min for 10 min to collect supernatants. Protein content of each sample was quantified through the BCA method. The samples were loaded in the same amount of protein. Primary antibody solution diluted at 1:1,000 was used to incubate the membranes at 4 °C overnight. Secondary antibody duiluted at 1:20,000 was added to the membranes. Images were acquired by Odyssey Western blot analyzer (LI-COR Biosciences).

### Statistics

Data were analyzed by SPSS 20.0 and expressed using mean ± standard deviation. While data were in accordance with normal distribution, one-way analysis of variance and *t*-test were performed for intergroup comparison by SO *vs* I/R, I/R *vs* LDP and I/R *vs* HDP. The intergroup comparison of HG was performed through multi-sample ranked data. *P* < 0.05 was taken as the criteria for determining the significance of differences.

## Results

### Neurological deficit scoring

CA1 region of hippocampus is highly related to learning ability (Jarrard, 1976). Based on related rat performance (Brambrink et al., 2006), neurological deficit scoring was given. Neurologic deficit scores were analyzed by *t*-test and *p* < 0.05 would be defined to be significant. The results showed that the SO group did not exhibit obvious neurological deficits, whereas the reperfusion group and rhodioloside groups presented severe neurological deficits after model construction and it gradually eased with time. The neurological deficits of the rhodioloside groups were significantly dose-dependently relieved after 24 h compared to those of the reperfusion group (Table 1).

**Table 1:** Comparison of neurological deficit scores at different time points.

Group	6 h	24 h	72 h	5 days
SO	3.16 ± 0.95	2.83 ± 0.68	2.00 ± 0.38	1.33 ± 0.30
I/R	33.67 ± 2.25^*^	29.50 ± 1.96^*^	23.17 ± 1.65^*^	19.50 ± 1.27^*^
LDP	31.35 ± 2.15^*^	21.67 ± 2.32^*^^,^^**^	15.83 ± 1.44^*^^,^^**^	12.17 ± 0.98^*^^,^^**^
HDP	30.67 ± 2.42^*^	18.00 ± 1.22^*^^,^^**^^,^^#^	11.50 ± 1.40^*^^,^^**^^,^^#^	9.67 ± 0.79^*^^,^^**^^,^^#^

**Notes.**

* *P* < 0.05 vs. SO.

** *P* < 0.05 vs. I/R.

# *P* < 0.05 vs. LDP.

### Pathological evaluation of brain tissues

**Figure 3:**
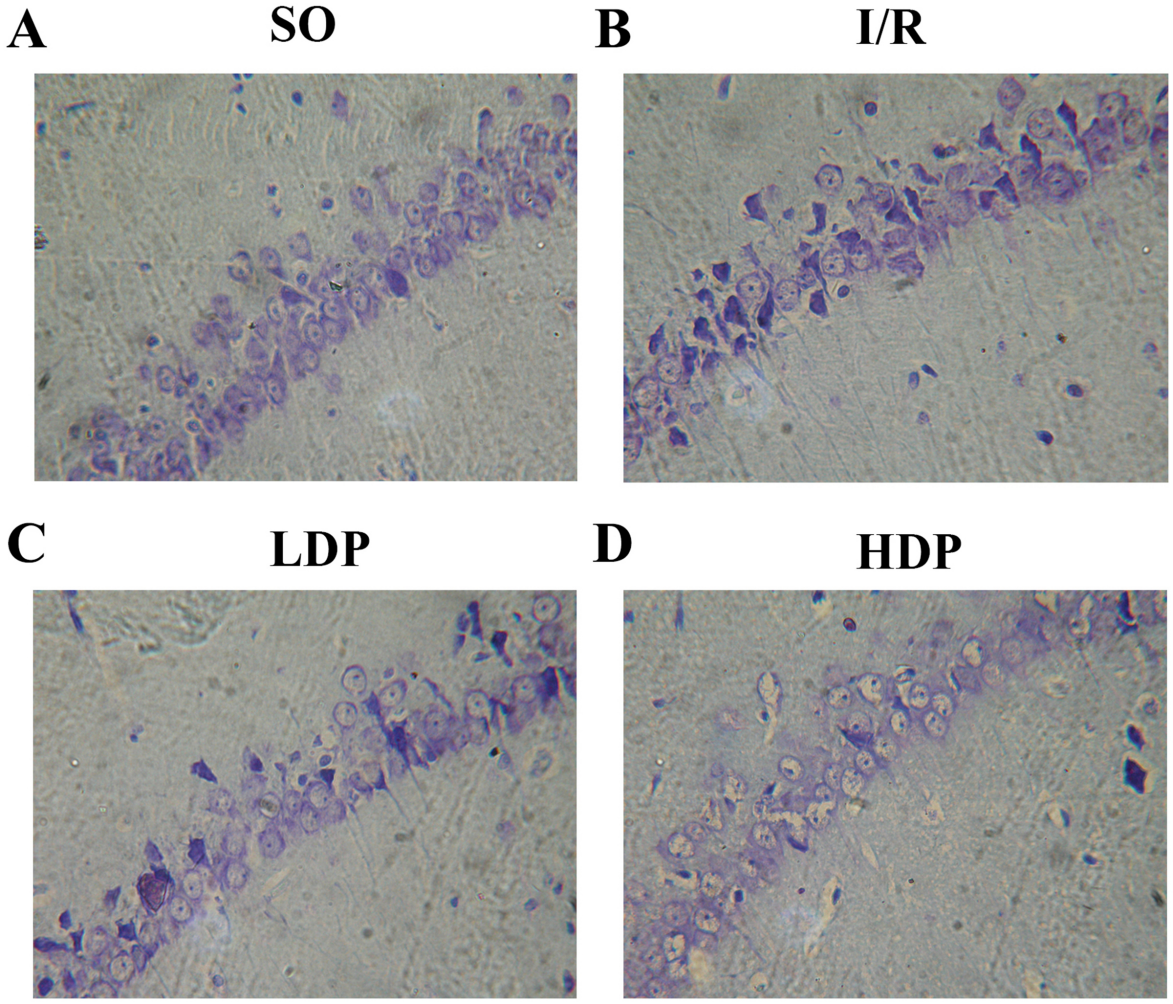
Thionine dyed histological slides of rat hippocampal CA1 neuron. Hippocampal CA1 cone cells showed in the SO group (A) arranged orderly with intact morphology and no deficiency; in the I/R group (B), cone cells became sparse and in disordered arrangement, and the shape turned triangular and irregular; in the LDP (C) and HDP (D) groups, the condition of neurons was remarkably improved.

The effects of rhodioloside on hippocampal CA1 neuron morphology in rats was evaluated by microscopy after thionine dyeing (Fig. 3). In the SO group, the hippocampal CA1 cone cells showed an orderly arrangement without deficiency, intact morphology and nuclei were large and circular in the centers of cells with clear nucleoli and abundant nissl bodies. When rats were subjected to total cerebral I/R, cone cells in hippocampal CA1 region became sparse and in disordered arrangement, and the size of residual cone cells are reduced with triangular and irregular shapes, accompanied with incomplete nuclear membrane and missing nuclei. Fortunately, the application of rhodioloside made a noteworthy improvement in the state of hippocampal CA1 neuron, especially in HDP group.

HG was given for each rat, and the number of rat in each group is shown in Table 2. ND and HG of rats was analyzed by *t*-test and *p* < 0.05 was set as a significant level. It is demonstrated in Table 2 that HG and surviving ND of SO group were 0–I and 181.17 ± 11.48 cells/mm respectively, representing a healthy state of tissue and ND. The I/R group showed severe tissue injury (HG grade II–III) and remarkable reduction in ND. However, rhodioloside groups saw better arrangement and cell morphology in hippocampal CA1 cone cells, lower HG and higher ND than those in the I/R group, especially in HDP group.

**Table 2:** HG and ND results for the hippocampal CA1 regions of rats in different groups.

Group	HG				ND (cells/mm)
	0	I	II	III	
SO	5	1	0	0	181.17 ± 6.59
I/R	0	0	2	4^*^	41.67 ± 3.77^*^
LDP	0	0	3	3^*^^,^^**^	58.33 ± 4.30^*^^,^^**^
HDP	0	1	3	2^*^^,^^**^^,^^#^	70.51 ± 5.46^*^^,^^**^^,^^#^

**Notes.**

* *P* < 0.05 vs. SO.

** *P* < 0.05 vs. I/R.

# *P* < 0.05 vs. LDP.

### Detection of neuronal apoptosis rate

**Figure 4:**
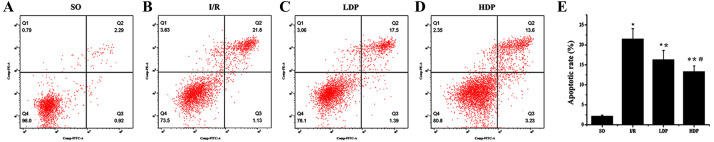
Hippocampal neuronal apoptosis rates for rats in different groups. (A–D) The apoptotic rate images obtained from flow cytometry. (E) Analytical results of apoptosis. Apoptotic rate in Q2 quadrant (late apoptosis) was analyzed by *t*-test method, and it shows that apoptosis was significantly increased in the I/R group while the LDP group reduced apoptosis and HDP saw an even lower apoptosis. ^∗^SO vs. I/R, *p* < 0.05; ^∗∗^I/R vs. LDP *p* < 0.05; ^#^LDP vs. HDP *p* < 0.05.

The results obtained from *t*-test analysis indicate that the hippocampal neuronal apoptotic rates of the I/R group were higher than the SO group. When rhodioloside was applied to I/R rats, apoptosis rates were decreased significantly and the apoptisis in HDP group was even less than in LDP group (Fig. 4).

### Expression of p53 in the CA1 region of the rat hippocampus

**Figure 5:**
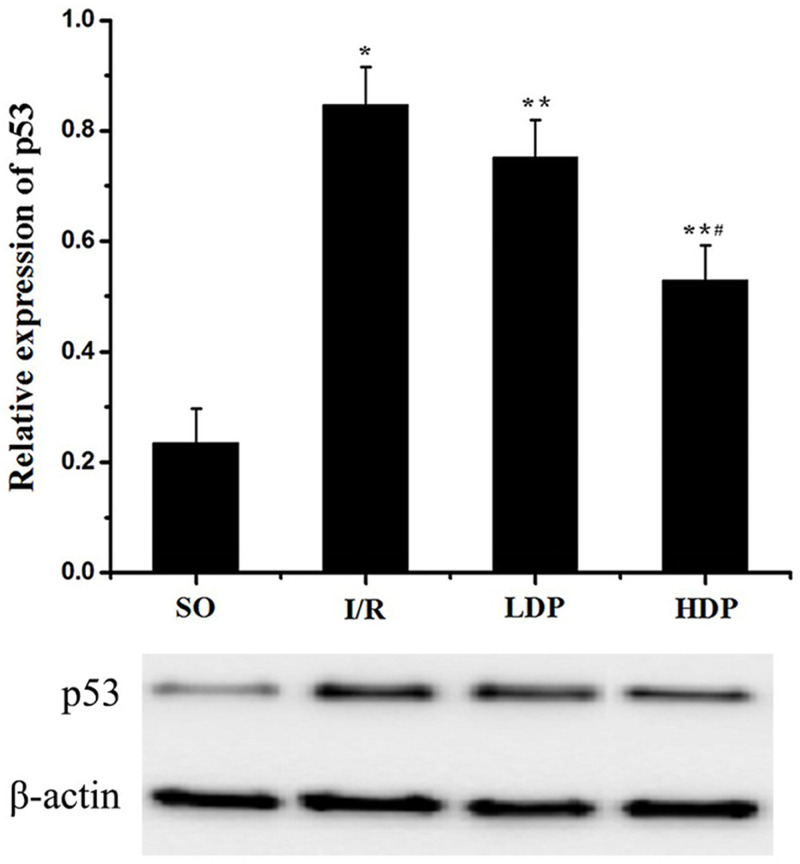
Expression of p53 protein in the CA1 region of the rat hippocampus. The expression of p53 was normalized to β-actin expression bands and analyzed by t-test to evaluate the significance. The results show p53 expression was increased in I/R group but the increase was lessened by rhodioloside in a dose-dependent pattern. ^∗^SO vs. I/R, *p* < 0.05; ^∗∗^I/R vs. LDP *p* < 0.05; ^#^LDP vs. HDP *p* < 0.05.

The gray value of p53 bands was analyzed by *t*-test to evaluate the significance (*p* < 0.05). The results indicate that expression of p53 was increased in the I/R group compared to the SO group, but in the rhodioloside treatment groups it was significantly lower than the I/R group and showed a dose-dependent pattern (Fig. 5).

### Expression of Bcl-2/Bax in the CA1 region of the rat hippocampus

**Figure 6:**
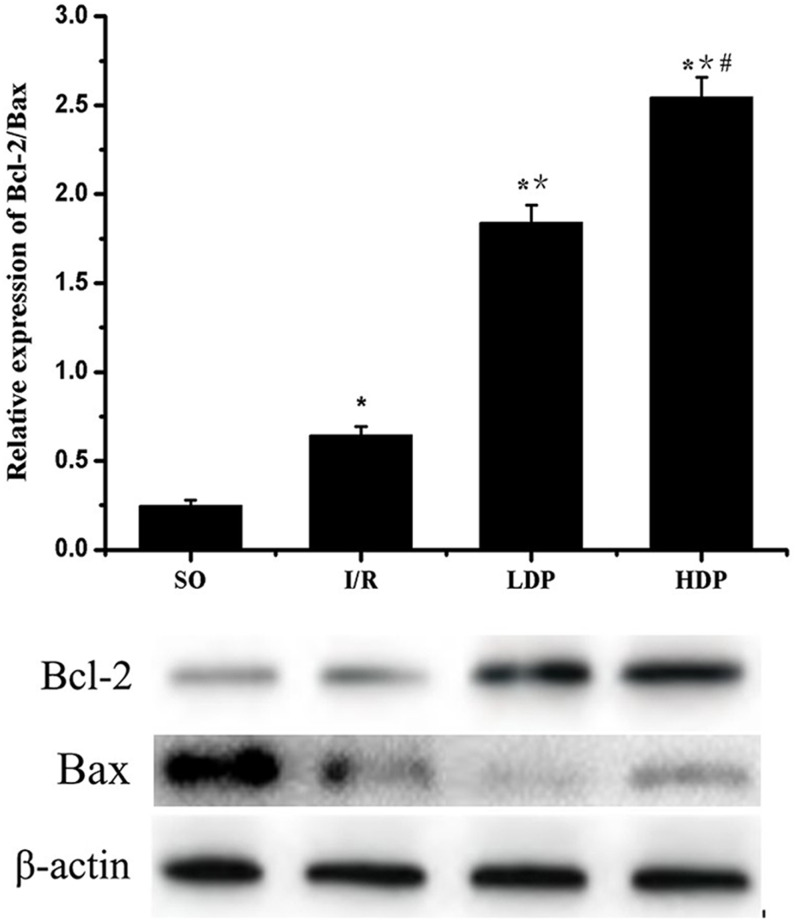
Expression of Bcl-2/Bax protein in the CA1 region of the rat hippocampus. The expression of Bcl-2 was normalized by Bax expression and analyzed by *t*-test. Bcl-2/Bax protein expression shows a slight increase in I/R group and a remarkable increase in the rhodioloside treatment groups (LDP group and HDP group) in a dose-dependent pattern. ^∗^SO vs. I/R, *p* < 0.05; ^∗∗^I/R vs. LDP *p* < 0.05; ^#^LDP vs. HDP *p* < 0.05.

The gray value of Bcl-2 and Bax bands was analyzed by *t*-test, and *p* < 0.05 was set as significance. It is shown in Fig. 6 that Bcl-2/Bax protein expression slightly increased in the I/R group compared to SO group and significantly increased in the rhodioloside treatment groups in a dose-dependent pattern.

## Discussion

Cerebral ischemia is a common factor of cerebral apoplexy. It makes multiple neuronal genes express abnormally, changes functional metabolism, and induces irreversible and intensive injury of cerebral functions in multiple pathological and physiological processes (Lee et al., 2018). Apoptosis is one of classical neuron pathways (Yuan & Yankner, 2000), therefore, studying the apoptosis-based mechanism of cerebral ischemic injury is vital.

Rhodioloside, a principal constituent of *R. rosea*, exerts a protective effect on the neural system (Khanum, Bawa & Singh, 2010), but its action mechanism has not been completely clarified and defined. Shi et al. (2012) previously compared the function of rhodioloside and an analog of rhodioloside (Tyrosol galactoside) on focal cerebral ischemia and studied their regulation to the expression of Bax. In this study, we constructed a more typical total cerebral I/R rat model, and studied the effects of total cerebral I/R and rhodioloside on rat performance, expression of p53 and the ratio of Bcl2 and Bax. The p53 protein is a P-containing, apoptosis-promoting nucleoprotein that consists of 393 amino acids, and normally expresses at low level and plays an important role in stabilizing and restoring chromosomal DNA (Meschini et al., 2010). However, when in nagative circumstances, such as ischemia and anoxia, chromosomal DNA structure would be injured; the protein kinase cascade reaction and NF-κB signaling pathway would be activated; and the transcription and translation of p53, would be up-regulated (Ci Ji et al., 2013; García-Ospina et al., 2003; Szczurek et al., 2017). As a transcription factor, p53 would further affect the expression of its downstream genes and promote cell cycle arrest to provide sufficient time for DNA repair. If DNA repair fails, p53 could induce neurons to apoptosis through transcription-dependent and nontranscription-dependent pathways (Yin et al., 1992). P53-promoted apoptosis processes mainly include actions on the Bcl-2 family and the Fas death receptor (Chou, Yang & Lee, 2015). Therefore, the discovery of neuroprotective drugs that block neuronal apoptosis pathways, up-regulate the expression of apoptosis-resistant proteins, and increase neuronal survival rates are necessary to develop strategies for the prevention and treatment of total cerebral ischemic injury.

In this experiment, we detected the apoptosis of hippocampal neuron, and found rhodioloside alleviated the apoptotic rate, which indicates the protective function of rhodioloside to neuron. p53, Bcl-2 and Bax are essential proteins to play a neuroprotective role in cerebral I/R by participating in apoptotic regulation (Cui et al., 2013; Levine, 1997). Therefore, the expression of total p53 protein and Bcl-2/Bax were measured to declare the anti-apoptotic pathways.

The results show that rhodioloside down-regulated p53 expression, and up-regulated Bcl-2/Bax, which primarily explained the mechanism of anti-apoptotic effects of rhodioloside. Interestingly, the expression of both p53 and Bcl-2/Bax was increased in I/R group, which implies that rat could arouse anti-apoptotic response to I/R induced apoptosis to achieve a balance, but the safeguard is weaker compared to the injury, showing increased apoptotic rate in I/R group in the end. In addition, reduced neurological deficit scores and increased neuronal survival rate of rats with total cerebral ischemia/reperfusion injury verify that rhodioloside has a neuroprotective effect against total cerebral ischemia/reperfusion injury by blocking neuronal apoptosis pathways and inhibiting neuronal apoptosis through interfering with p53 and Bcl-2/Bax protein expression. Rhodioloside has been reported to play a neuroprotective role through different approaches, such as activation of PI3K/Akt signaling (Wei et al., 2017; Zuo et al., 2018), inhibition of complement (Lai et al., 2018), suppression of receptor-interacting protein 140 (Chen et al., 2016), inhibition of inflammation (Wei et al., 2017; Zhang et al., 2019; Zhou et al., 2019) etc. Based on these findings, our study provides more evidence for the neuroprotection of rhodioloside. Besides, rhodioloside may also involve in other approaches to achieve its function on protecting neuro. Thus, other pathways in neuroprotective effects of rhodioloside needs further study. Plus, a combined treatment should be considered to improve the effectiveness of rhodioloside.

## Conclusion

Rhodioloside achieves its function of neuroprotection through several approaches, and the role of rhodioloside in rats with cerebral ischemia/reperfusion brain injury is related to preventing neuros from apoptosis through increasing Bcl-2 protein level and reducing p53 protein level.

##  Supplemental Information

10.7717/peerj.10056/supp-1 Supplemental Information 1 Western blot original image

10.7717/peerj.10056/supp-2 Supplemental Information 2 Raw data: numerical value in Table 1 and numerical value of ND, apoptotic rate and western blot grey value

## Additional Information and Declarations

### Data Availability

The following information was supplied regarding data availability: Data are available as Supplemental Files.
