# Supplementary figures and images for: Effects of rhodioloside on the neurological functions of rats with total cerebral ischemia/reperfusion and cone neuron injury in the hippocampal CA1 region
Source: PeerJ. 2020 Nov 9;8:e10056. doi: 10.7717/peerj.10056 (PMC7659626; doi:10.7717/peerj.10056)

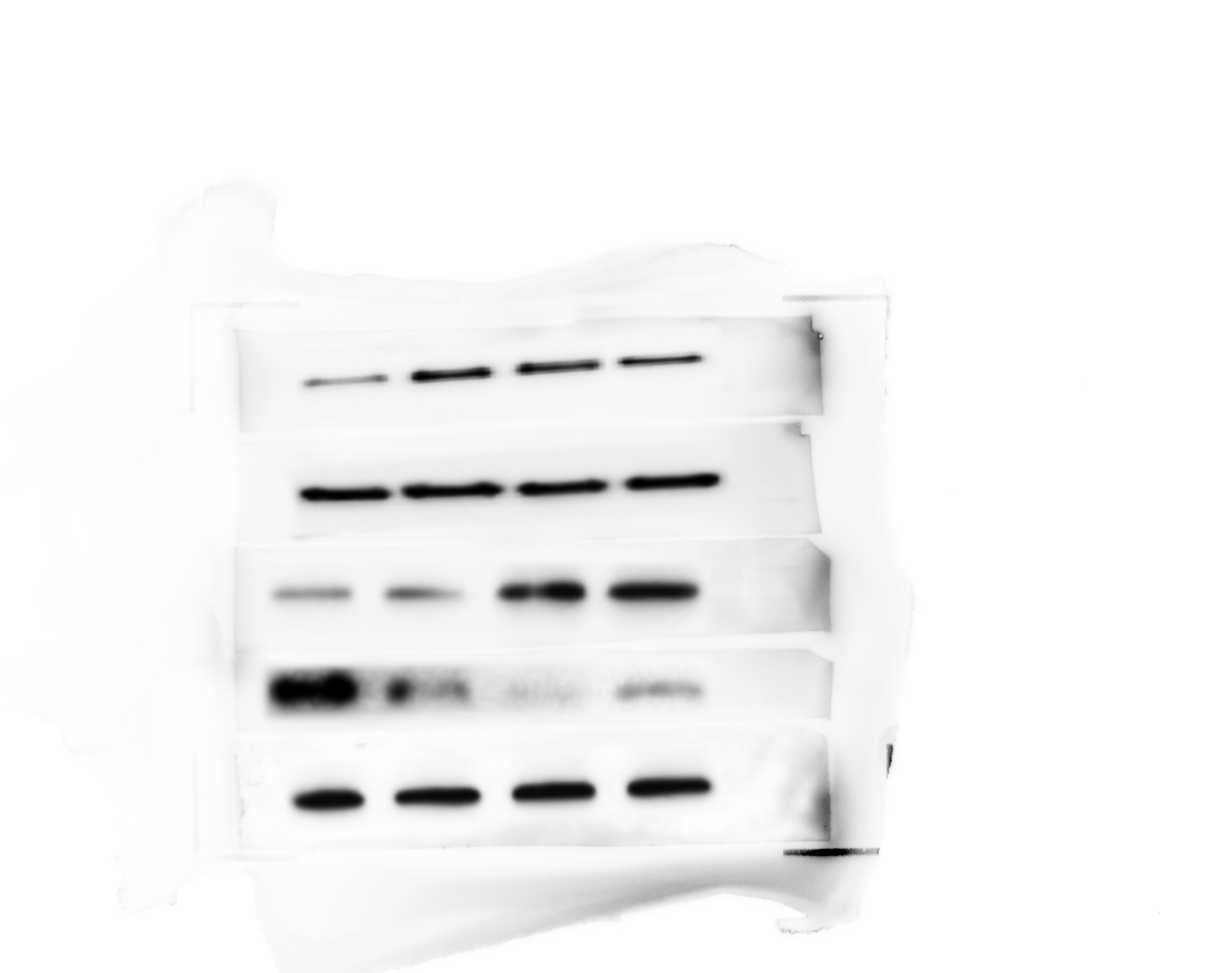

Supplement: Supplemental Information 1 [file peerj-08-10056-s001.jpg]
